# Inhibition of EED enhances osteogenic differentiation and bone formation: a potential therapeutic strategy for osteogenesis imperfecta

**DOI:** 10.1093/jbmrpl/ziag142

**Published:** 2026-08-22

**Authors:** Margarita E Carrasco Jeldres, Oksana Pichurin, Samantha R Weaver, Eric Cheang, Leah E Tokach, Jennifer J Westendorf, David R Deyle

**Affiliations:** Department of Clinical Genomics, Mayo Clinic, Rochester, MN 55905, United States; Department of Clinical Genomics, Mayo Clinic, Rochester, MN 55905, United States; Department of Comparative Biosciences, University of Wisconsin-Madison, Madison, WI 53706, United States; Department of Clinical Genomics, Mayo Clinic, Rochester, MN 55905, United States; Department of Clinical Genomics, Mayo Clinic, Rochester, MN 55905, United States; Department of Orthopedic Surgery, Mayo Clinic, Rochester, MN 55905, United States; Department of Biochemistry and Molecular Biology, Mayo Clinic, Rochester, MN 55905, United States; Department of Clinical Genomics, Mayo Clinic, Rochester, MN 55905, United States; Department of Molecular Medicine, Mayo Clinic, Rochester, MN 55905, United States

**Keywords:** ER stress, EZH1, EZH2, ISR, OI, osteoblasts

## Abstract

Osteogenesis imperfecta (OI) is a heterogeneous group of inherited connective tissue disorders primarily caused by dominant mutations in *COL1A1* or *COL1A2* that impair type I procollagen folding and secretion. Misfolded collagen accumulates in the endoplasmic reticulum (ER), triggering ER stress and osteoblast dysfunction, and bone fragility. Current pharmacologic therapy focuses on inhibiting bone resorption but has limited efficacy and does not address the underlying biology of the disease. The epigenetic regulator polycomb-repressive complex 2 (PRC2) has emerged as an important regulator of bone formation. Genetic and pharmacologic disruption of PRC2 enhanced osteogenic differentiation in WT cells. Here, we demonstrate that inhibition of the PRC2 through targeting its essential component embryonic ectoderm development (EED) enhances osteogenic differentiation, improves bone architecture in male *Col1a2*^+/G610C^ OI mouse models, modulates the integrated stress response (ISR), and improves ER morphology in OI cells. These findings identify EED inhibition as a novel epigenetic strategy to restore collagen homeostasis and improve skeletal integrity in OI.

## Introduction

Osteogenesis imperfecta (OI) encompasses a clinically variable group of heritable disorders characterized by bone fragility and extra-skeletal manifestations including dentinogenesis imperfecta, hearing loss, and blue sclerae.^1–4^ Most cases result from glycine substitutions in *COL1A1* or *COL1A2*, disrupting triple-helix formation within type I collagen.^1–4^ These mutations impair hydrogen bonding, delay procollagen folding in osteoblasts, cause intracellular accumulation, and reduce collagen secretion in bone.^5–7^ In OI osteoblasts, misfolded collagen activates endoplasmic reticulum (ER) stress pathways, including the integrated stress response (ISR) and unfolded protein response (UPR), contributing to apoptosis and impaired bone formation.^5–8^ Even under physiologic conditions, a portion of procollagen is misfolded and degraded,^9–13^ and pathogenic variants exacerbate this process through altered ER-to-Golgi trafficking and autophagic rerouting.^13–16^

Current treatments focus on fracture prevention rather than correcting collagen biosynthesis. Bisphosphonates increase BMD but have inconsistent functional benefits.^17–20^ Other agents, including denosumab and anti-sclerostin antibodies, show potential but remain limited in durability or evidence of increased bone strength.^21^^,^^22^ Rapamycin and 4-phenylbutyric acid (4-PBA) have been explored for modulating ER stress, but improvements in bone strength remain modest or inconsistent.^23–25^ Thus, targeting intracellular collagen processing represents a promising therapeutic direction.

Epigenetic regulation plays a central role in osteoblast differentiation.^26–28^ PRC2, composed of embryonic ectoderm development (EED), SUZ12, Rbbp4, and catalytic subunits EZH1 or EZH2, mediates H3K27 trimethylation to control chromatin structure and gene expression.^29–33^ Prior studies demonstrate that EZH2 inhibition stimulates bone formation.^34–37^ Embryonic ectoderm development is essential for PRC2 activity via binding to H3K27me3 and promoting allosteric activation.^33^ We hypothesized that EED inhibition would enhance collagen production and bone formation in an OI model.

## Materials and methods

### Mouse models

The *Col1a2*^+/G610C^ knock-in mouse model of OI (aka, G610) was kindly provided by Dr. Christina Jacobsen.^6^ The Mayo Clinic Institutional Animal Care and Use Committee approved all experiments, and work was completed according to guidelines of the National Institutes of Health and the Institute of Laboratory Animal Resources, National Research Council. Animals were housed in an accredited facility with 12-h light/dark cycles and supplied food and water ad libitum (Pico-Lab Rodent Diet 20, LabDiet).

### Isolation of primary mouse bone-derived osteoblasts from tibias

Osteoblasts were isolated from tibias Col1a2^+/G610C^ mice as previously described.^38^ All procedures were under sterile condition. Briefly, the tibias were collected and cleaned. The epiphyses were cut and bones flushed several times with medium to deplete blood cells. Tibias were cut into 1 to 2 mm pieces. Bone chips were digested with 0.1% w/v Collagenase I (Sigma) for 90 min in the shaking incubator at 37 °C with shaking speed of 200 g. Digestion medium was then removed, and the bone chips were rinsed with medium 3 times. Medium was added to the bone chips and incubated at 37 °C for 3-4 d. Medium was changed every 3-4 d until cells were 70%-80% confluent.

### Cell maintenance media and osteogenic differentiation

MC3T3-E1 subclone 4 cells (referred to as MC3T3 cells) and mouse osteoblasts were maintained and expanded in ascorbic acid-free α-MEM supplemented with 10% FBS (R&D Systems) and 100 units/mL penicillin (Gibco). Cells were plated at a density of 10 000 cells/cm^2^ on standard tissue culture plates (Corning) in maintenance medium. To induce osteogenic differentiation, maintenance medium was replaced with osteogenic medium which consisted of α-MEM supplemented with 10% FBS, 100 units/mL penicillin, 50 μg/mL ascorbic acid (Sigma), and 10 mM beta-glycerol phosphate (Sigma). Osteogenic medium was replaced every 3 d. Vehicle (dimethyl sulfoxide [DMSO]), A-395 or MAK683 were added at intervals described. Human OI bone marrow stromal cells (BMSCs) hOIMSC10 and hOIMSC11 lines were established from discarded bone fragments of affected individuals undergoing corrective surgery with institutional review board approval, and the mutations in these OI MSC lines were identified as described previously.^39^ Osteogenesis imperfecta MSCs were grown at 37 °C in MSC medium consisting of Dulbecco’s modified Eagle’s medium with low glucose containing 10% characterized fetal bovine serum (HyClone Laboratories), 100 U/mL penicillin, 100 μg/mL streptomycin, and supplemented with 2 mmol/L-glutamine. OI MSCs were induced to form bone using osteogenic induction medium consisting of MSC medium plus dexamethasone, ascorbic acid, and β-glycerol phosphate as described.^40^

### Assessments of in vitro osteogenic differentiation

To evaluate the osteogenic differentiation of cultured cells, alkaline phosphatase (ALP) activity was evaluated at 7 d using the ALP assay kit (Sigma-Aldrich) according to the manufacturer’s instructions. Mineral deposits on the cultured cells were visualized at the indicated time points, medium was removed, and cells were washed twice with PBS. After washing, cells were fixed in 10% neutral-buffered formalin for 1 h. Neutral-buffered formalin was then removed, cells were washed with PBS, and stained with 2% alizarin red pH 4.2 (Thermo Fischer Scientific) for 10 min. The stain was removed, and cells were washed five times with water. Images of the wells were taken, and staining was quantified using ImageJ program (https://imagej.nih.gov/ij/index.html).

### RNA isolation and quantitative real-time reverse transcription PCR

Cells were lysed, and RNA was extracted using TRI-Reagent (Zymo Research). RNA was isolated using DirectZol RNA MiniPrep (Zymo Research). Isolated RNA was quantified, and quality was tested using NanoDrop 2000 spectrophotometer (Thermo Fischer Scientific). RNA was reverse transcribed into complementary DNA using a reverse transcription kit and protocol (Promega). Gene expression was quantified using RT-qPCR; each reaction was performed with 3.3 ng of complementary DNA, 0.08 μM of primer pairs, and 1× QuantiTect SYBR Green PCR Kit (Qiagen) using the CFX384 Real-Time System (Bio-Rad). Transcript levels were analyzed by the 2^−ΔΔCt^ method and normalized to the housekeeping gene GAPDH. The primers used are described in Table 1.

**Table 1:** PCR primers.

Gene	Forward primer	Reverse primer
**Bglap3**	GCACACCTAGCAGACACCAT	ACTGAAGCTCCAAGGTAGCG
**Phex**	CAACGTTCCGCGGTCAATAC	ACAGCCAGAGAGAGTGTTGC
**Phospho1**	CACATCATCCACAGTCCCTCG	CTGTTGGTCTCCAGCTGTCAT
**Hspa9**	CAAGGGTGCAGTGGTTGGTA	GGGGTAGTTCTGGCACCTTC
**Atf5**	GGCTGGCTCGTAGACTATGG	ACCCGCTCAGTCATCCAATC
**Ddit3**	CCTGAGGAGAGAGTGTTCCAG	GACACCGTCTCCAAGGTGAA
**Eif3c**	GGAACTACGGCAAACAGCCT	TGGCATTACGGATGGTTCGT
**Eif4ebp1**	ACTCACCTGTGGCCAAAACA	TTGTGACTCTTCACCGCCTG
**Nupr1**	GGTCGGACCAAGAGAGAAGC	GTGGTCTGGCCTTATCTCCA
**Atf4**	TGCCGGTTTAAGTTGTGTGC	GGATTTCGTGAAGAGCGCCAT
**Atf6**	TGGAAGCCTAAAGAGGACCTG	CGTGGGAGGACAGAGAAACAA
**Ern1**	CTCCTCTGTCTGCATCCACC	GATACGGTGGTCGGTGTGTT
**Eif2ak3**	TTTTCCATCCTCAGCCCCACAG	ACCACGACCCATGCACTGA

### Bone phenotyping

Micro-CT (μCT) was performed on femora using a SkyScan 1276 system (Bruker). Samples were scanned at 55 kV and 200 μA with a 10 μm isotropic voxel size, using 0.2° rotation steps over 360°, frame averaging of 4, and a 0.25 mm Al filter. Projection images were reconstructed into 2D cross-sections using NRecon software (Bruker) with beam hardening and post-alignment corrections applied. 3D reconstructed datasets were reoriented to align the femoral longitudinal axis vertically prior to analysis. Trabecular bone microarchitecture was quantified using CtAN software (Bruker) within a defined ROI corresponding to 5% of the femur length, positioned in the metaphysis at a standardized distance proximal to the distal growth plate. Trabecular bone was segmented using a global gray-value threshold of 50.

### Bone dimensions and 3-point bending

Bone dimensions were measured with a digital caliper (Digimatic Absolute, Mitutoyo) capturing the total length of the femur, the medial-lateral diameter at the mid-shaft, a, and the anterior–posterior dimension at the mid-shaft, b. A 3-point bend fixture was mounted on a servohydrauilic mechanical testing frame (Model 858, MTS Systems) instrumented with a 25-lb capacity load cell (Model MDB-25, Transducer Techniques). Femurs were mounted on supports spanning 8 mm. Point loading was applied to the femur midshaft allowing the bones to flex about an axis aligned with the medial-lateral line. Specimens were kept moist by irrigating them with phosphate buffered saline solution. Loading was applied under displacement control at a rate of 20 mm/min until fracture. Force and displacement data was sampled at 256 Hz. The peak load was quantified as was the peak displacement. The stiffness was calculated from the slope of the linear region of the force displacement curve. All failures occurred at the femur midshaft below the point of load application.

### Electron microscopy

Osteogenesis imperfecta BMSCs were plated on a coverslip and allowed to attach overnight. The next day, the medium was changed to contain the EED inhibitor A-395, and the cells were grown for 7 d. Cells were fixed and then embedded in epoxy resin and sectioned. Transmission electron microscopy was performed.

### Statistical analysis

Experiments were performed using biological triplicates from cultures of MC3T3 cells. The results of the experiments are presented as mean ± SD. Statistical analysis was performed using one-way ANOVA. Statistical analysis was performed using GraphPad Prism Software version 8.4.2 (https://www.graphpad.com/).

## Results

### E‌ED inhibition promotes osteogenic differentiation

Inhibiting both EZH1 and EZH2 with a single small molecule has not been possible to date. Another essential core protein of the PRC2 is EED. Embryonic ectoderm development does not have methyltransferase activity like EZH1 or EZH2 but directly interacts with these proteins to enhance their activity.^33^ In addition, EED binds H3K27me3 through its aromatic cage residues, thereby promoting the allosteric activation of PRC2 and propagating H3K27me3 of neighboring histones.^33^ Loss of EED abolishes the activity of PRC2 and does affect bone development.^34^^,^^35^

**Figure 1:**
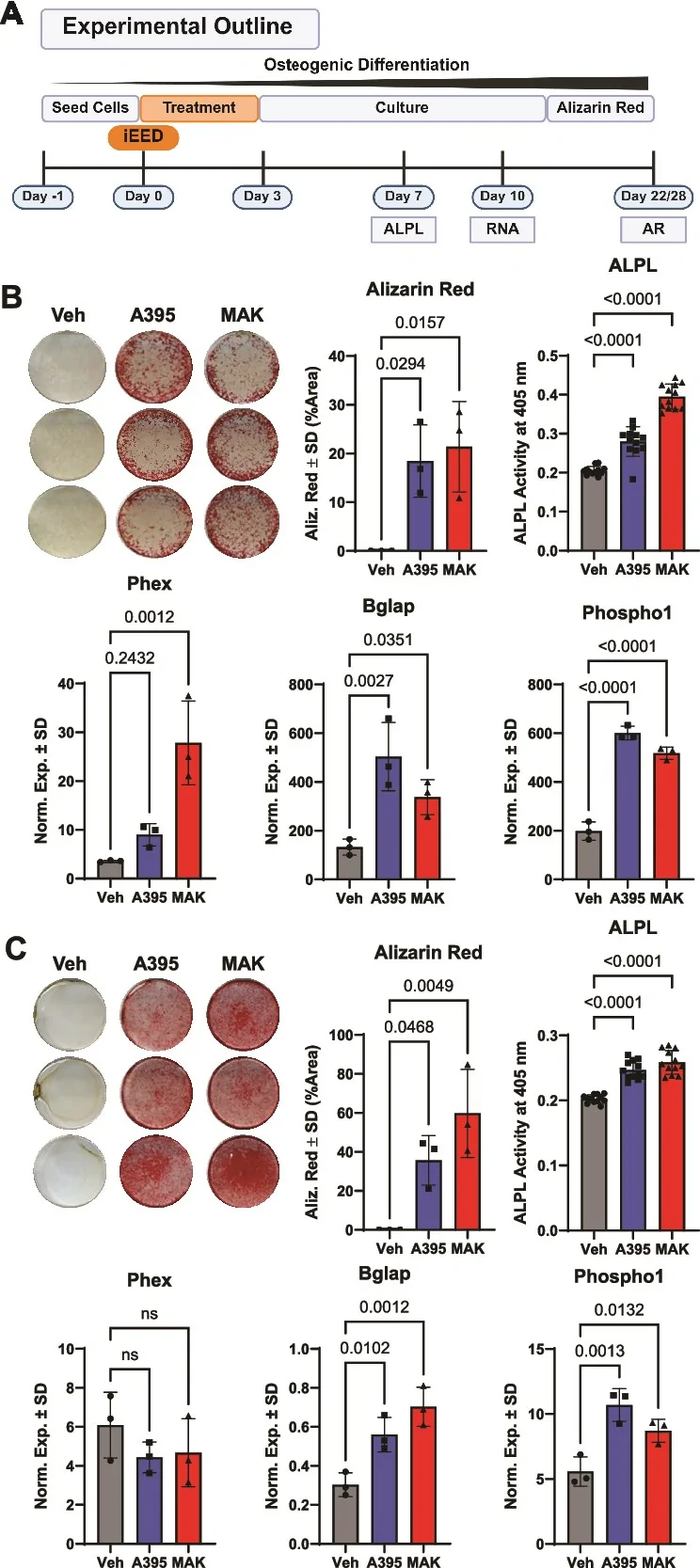
Embryonic ectoderm development (EED) inhibitors stimulate osteogenesis. Experimental scheme for adding EED inhibitors, A-397 or MAK to osteogenic differentiation assays (iEED) (A). Alizarin red staining, ALPL activity and qPCR for osteogenic genes expressed in differentiating MC3T3 cells (B) or differentiating murine primary osteoblasts (C) treated with EED inhibitors A-395 and MAK683. Comparisons analyzed using one-way ANOVA. *p*-values are shown.

We chose to assess EED inhibition of bone differentiation in vitro using 2 compounds, A-395 and MAK683, that have been shown to reduce H3K27me3.^41^^,^^42^ To evaluate their effects on osteogenesis, we first exposed MC3T3 cells to 5 μM A-395 or MAK683 for the initial 3 d of a 22-d osteoblast differentiation protocol, with RNA and protein collections and alizarin staining performed at designated intervals **(**Figure 1A**)**. A-395 or MAK683 induced expression of several RNA transcripts associated with bone mineralization, including Bglap, Phex, and Phospho1 (Figure 1B). A-395 and MAK683 enhanced calcium deposition in osteogenic medium. There was also an increase in ALPL activity in A-395 and MAK683 treated cells. Similar to MC3T3 cells, BMSCs isolated from the *Col1a2*^+/G610C^ mouse and exposed to 5 μM A-395 or MAK683 showed enhanced in vitro osteogenic differentiation and osteogenic genes (Figure 1C). Taken together, the disruption of the PRC2 by inhibiting EED with A-395 or MAK683 increased osteogenic differentiation in MC3T3 cells and OI BMSCs.

### Dose-dependent effects of EED inhibitors in OI mice

**Figure 2:**
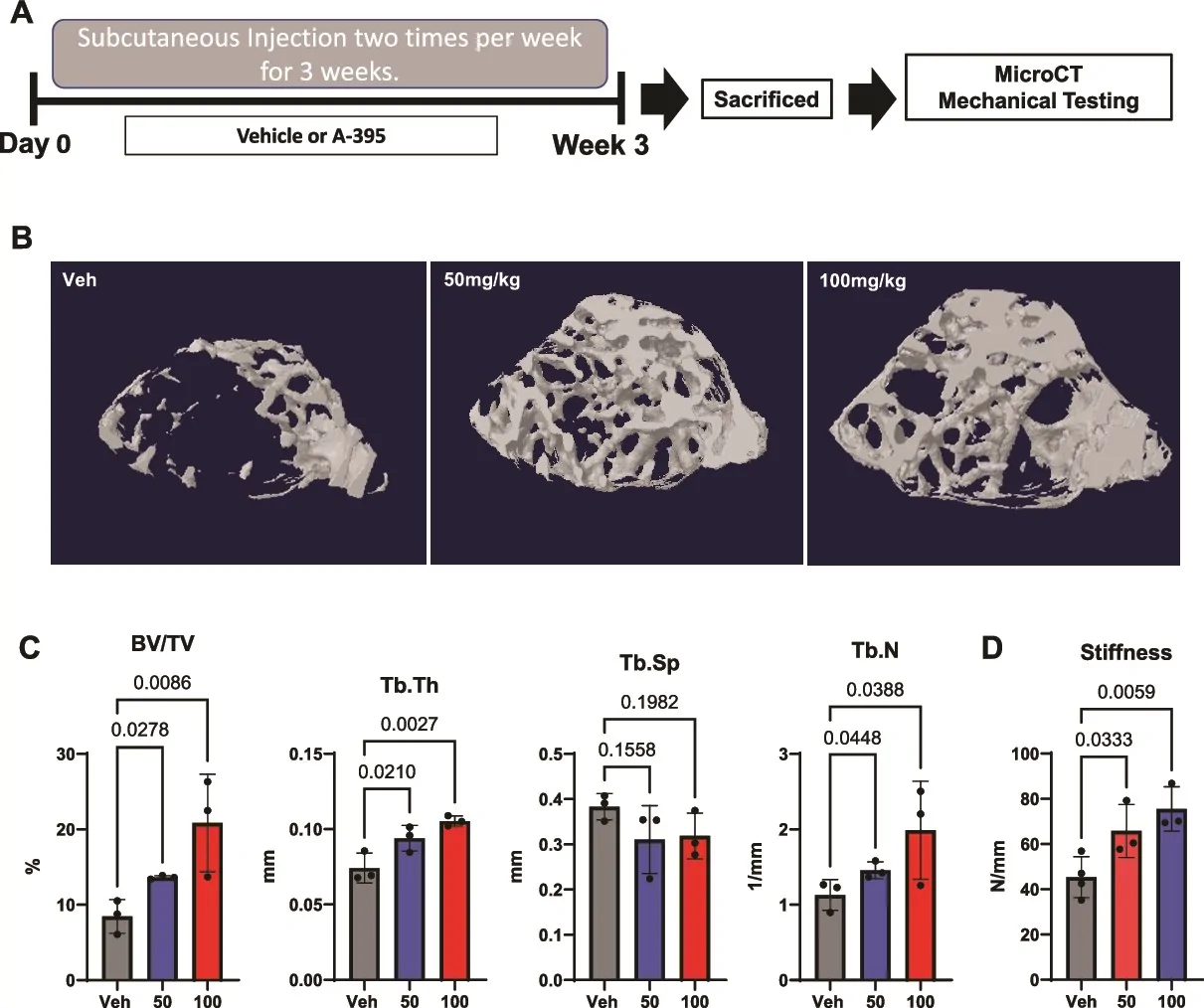
Embryonic ectoderm development (EED) inhibition improves bone architecture and stiffness in G610C mice. Experimental design for in vivo testing of A-395 (A). Micro-CT reconstructions of trabecular bone density in distal femurs after A-395 treatments (B). Micro-CT assessment of bone structural properties of G610C male mice after treatment with A-395 (C). Three-point bending showed increased stiffness in A-395 treated animals **(**D). Comparisons analyzed using one-way ANOVA. *p*-values are shown.

To determine the effects of EED inhibition on bone density in vivo, G610C mice were injected with A-395. We initially injected 3 5-wk-old male G610C mice with either 300 mg/kg A-395 or DMSO control twice a week by s.c. injection for 3 wk because this concentration was used previously in mouse models of diffuse large B-cell lymphoma.^41^ These mice experienced weight loss and showed signs of poor health (data not shown). We chose to reduce the dose range to include 50 and 100 mg/kg A-395 (Figure 2A). Mice receiving either 50 mg/kg or 100 mg/kg A-395 twice a week by s.c. injection for 3 wk maintained better overall health and had improved bone architecture (Figure 2B). We found that both lower doses improved percent bone volume, trabecular thickness, and trabecular number in OI mice (Figure 2C). Also, 3-point bending showed improved stiffness in both the 50 and 100 mg/kg treatment groups (Figure 2D, Table 2).

**Table 2:** Mouse 3-point bending measurements.

Specimen	Weight (gm)	a: ML (mm)	b: AP (mm)	Length (mm)	Span (mm)	Max load (N)	Max deflection (mm)	Stiffness (N/mm)
**Veh**	25.4	1.68	1.18	14.5	8	11.3	0.34	47.0
**Veh**	25.2	1.81	1.21	15.0	8	15.7	0.59	35.2
**Veh**	28.4	2.06	1.38	15.6	8	20.6	0.59	42.5
**Veh**	25.7	1.6	1.25	14.82	8	13.44	0.32	56.85
**50**	35	1.63	1.17	15	8	15.47	0.35	60.42
**50**	28.4	1.75	1.27	15.07	8	19.38	0.33	79.22
**50**	26.4	1.97	1.33	15.03	8	19.94	0.48	57.72
**100**	26.5	1.78	1.32	15.28	8	19.11	0.39	70.10
**100**	28	1.8	1.25	15	8	20.49	0.39	86.83
**100**	26.5	1.91	1.26	15.26	8	18.14	0.39	69.43

### E‌ED inhibition modulates ISR but not canonical UPR

**Figure 3:**
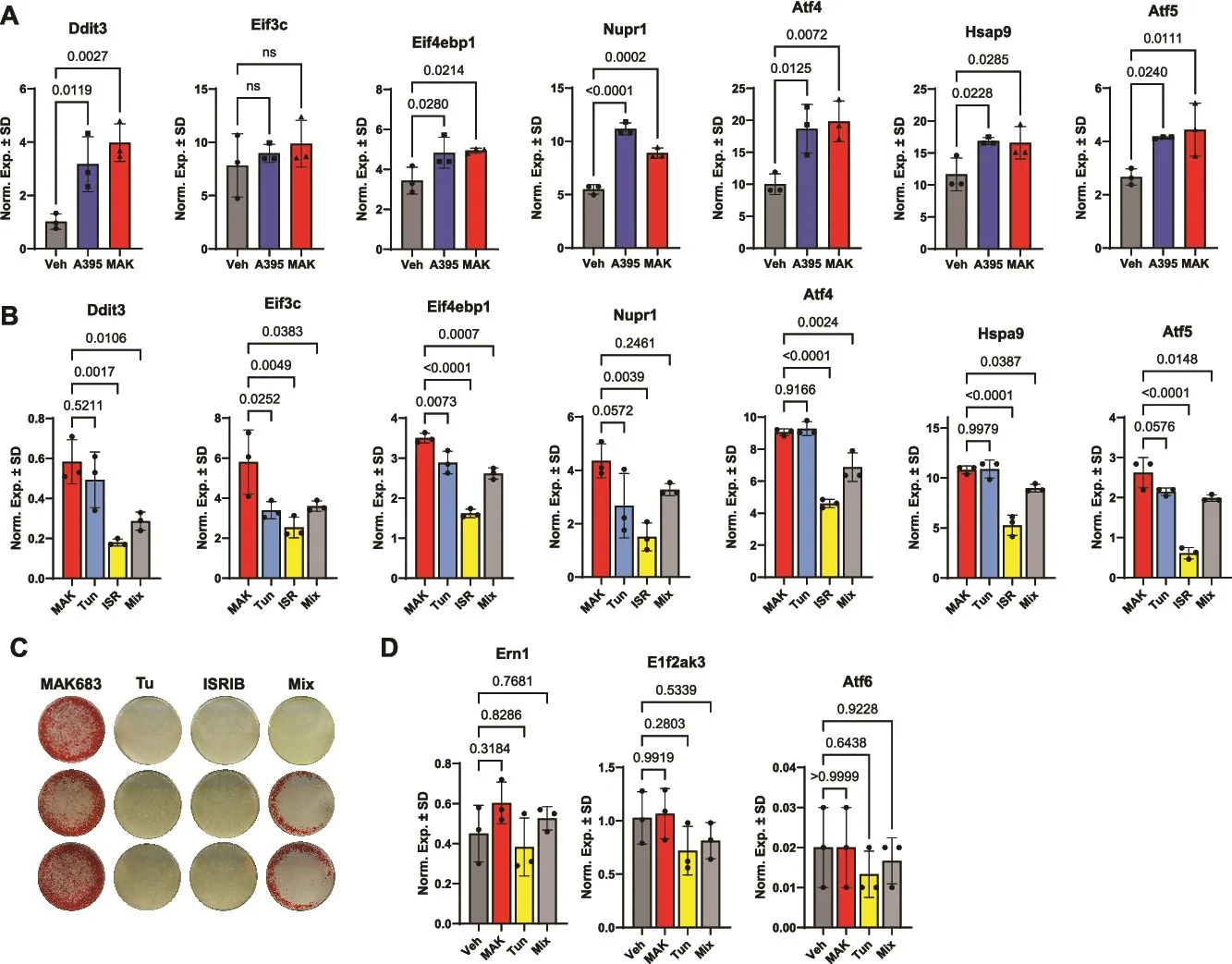
Embryonic ectoderm development (EED) inhibition alters integrated stress response (ISR) gene expression. qPCR data of MC3T3 cells treated with EED inhibitors A395 and MAK683 showing an increase in ISR gene expression compared to vehicle control (Veh) (A). Inhibition of the ISR pathway abolishes the increase in ISR gene expression (B) and in vitro bone formation (C) due to EED inhibitor treatment. Embryonic ectoderm development or ISR inhibition does not alter unfolded protein response (UPR) gene expression (D). Comparisons analyzed using one-way ANOVA. *p*-values are shown.

Recently, it has been shown that that genes involved in the ISR pathway are upregulated in G610C OI mice.^6^ To determine if PRC2 loss alters the ISR pathway, we incubated MC3T3 cells with 5 μM A-395 or MAK683 for 4 d and then collected RNA at day 10. Gene expression analysis showed that EED inhibitors increased the expression of most ISR genes (Figure 3A). We next determined if the ISR pathway was integral to PRC2 regulation of osteogenic differentiation. MC3T3 cells were cultured in the presence of the 5 μM MAK683, tunicamycin to induce the ISR pathway, the ISR inhibitor (ISRIB), or a cocktail of all 3 molecules and then assessed changes in gene expression. ISRIB lowered ISR gene expression in cells with and without EED inhibition (Figure 3B). In addition, ISRIB abrogated the increased osteogenic differentiation seen with the EED inhibitor (Figure 3C). No change in genes associated with UPR pathway were observed after treating cells with an EED inhibitor or after treating cells with ISRIB (Figure 3D) suggesting selective ISR modulation.

### E‌ED inhibition in human OI cells

**Figure 4:**
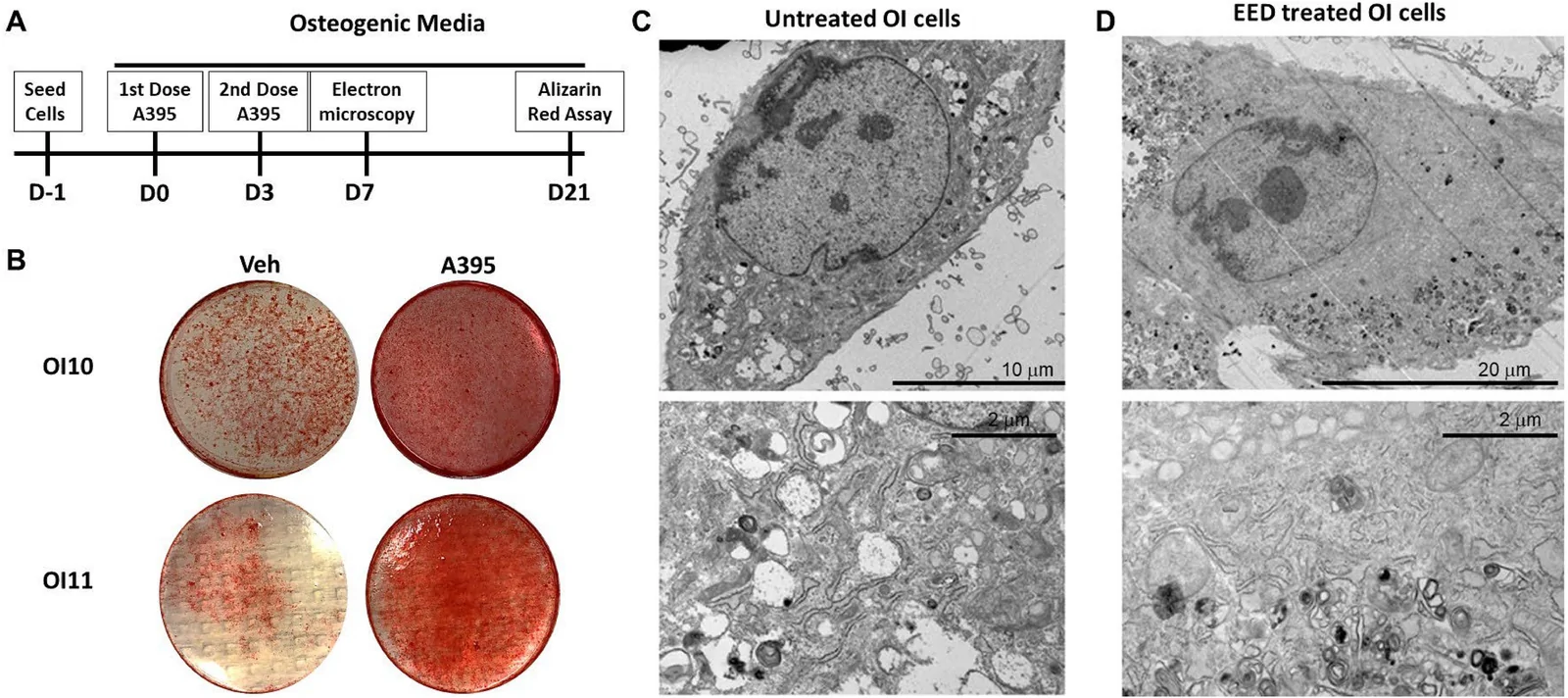
Embryonic ectoderm development (EED) inhibition in human OI cells. Experimental scheme for EED inhibition and osteogenic differentiation is shown (A). Human OIMSC10 (OI10) and hOIMSC11 (OI11) cells show enhanced osteogenic differentiation after treatment with A-395 and compared to vehicle control (Veh) (B). Transmission electron microscopy of OI10 cells showed that untreated cells exhibited ER enlargement and cellular vacuolization (C) which resolved with EED treatment (D).

To determine the effect of EED inhibition on human OI cells, we tested cells from our biobank of patient-derived, OI BMSCs. Human OIMSC10 harbors a *COL1A1* variant and hOIMSC11 harbors a *COL1A2* variant. A-395 enhanced osteogenic differentiation in both (Figure 4A and B). Collagen molecules retained in fibroblasts cause ER enlargement and activate the ER stress response.^5^^,^^43^ To examine whether PRC2 inhibition may be able to improve collagen processing, we treated our hOIMSC10 cell with either 5 μM A-395 or DMSO control. Transmission electron microscopy demonstrated that hOIMSC10 OI cells contained characteristic enlarged ER cisterns (Figure 4C). Osteogenesis imperfecta cells incubated with A-395 did not contain enlarged ER segments; however, more lamellar body-like structures were observed (Figure 4D). Lamellar body-like structures are secretory organelles and may represent increased collagen secretion, suggesting improved secretory homeostasis.

## Discussion

Treating OI remains a challenge as the genetic variants causing OI not only lead to abnormal bone matrix formation but also impaired osteoblastogenesis and cell death due increased intracellular stress. Previous studies have shown that the PRC2 regulates osteoblastogenesis and maturation that may be exploited to enhance bone formation.^34–37^ In this study, we demonstrated that inhibition of the PRC2, specifically through targeting the core component EED, is a novel strategy to enhance osteogenesis and improve bone properties in a murine model of OI. Pharmacologic inhibition of EED promotes bone formation, improves bone microarchitecture, and enhances mechanical strength, while also modulating cellular stress pathways linked to collagen misfolding in mouse and human osteoblastic cells.

Inhibition of EED in G610 mice resulted in increased bone mass supporting prior observations that PRC2 restrains osteogenic differentiation^29–33^ and importantly, suggests that PRC2 inhibition can improve the bone phenotype in OI. Our in vivo findings were supported by in vitro data showing that EED inhibitors (A-395 and MAK683) enhance osteogenic differentiation in MC3T3 cells, murine OI BMSCs, and patient-derived OI stromal cells. Together, these results indicate that PRC2 inhibition exerts a conserved pro-osteogenic effect.

Accumulation of misfolded procollagen proteins in the ER can trigger either the UPR through the eukaryotic translation initiation factor 2 alpha kinase 3 (PERK) branch or the ISR.^5–8^ Interestingly, mutations in *COL1A1* procollagens upregulate autophagy, while mutations in *COL1A2* procollagen only activate the auto-degradative response. Regardless of the mechanism, all OI fibroblasts underwent apoptosis.^25^ Because of these findings ER stress and autophagy are viable targets for OI treatment. Molecules that modulate these pathways in OI include rapamycin, an mTOR pathway inhibitor, and 4-PBA, a histone deacetylase (HDAC) inhibitor.^23^^,^^25^ While these molecules seem to improve bone density and trabecular bone formation, they have not been shown to improve bone strength and their effects varied dependent on the type of causative variant.^24^ In our study, we also observed improvements in bone architecture; however, the inhibition of EED also showed improvements in bone strength.

Our data also points to a possible link between EED and the ISR in osteoblasts. We found that EED inhibition selectively upregulated ISR-associated genes without activating canonical UPR pathways. Furthermore, pharmacologic inhibition of the ISR using ISRIB attenuated the pro-osteogenic effects of EED inhibition, indicating that ISR activation is not merely a byproduct but a functional mediator of enhanced differentiation. This finding is notable given prior reports that ISR activation is elevated in the G610C model.^6^ Our results suggest a more nuanced role in which controlled ISR activation may support osteoblast function under conditions of proteotoxic stress.

We also examined the intracellular effect of EED inhibition on OI cells. Transmission electron microscopy suggests that EED inhibition reduces ER dilation in OI cells, a hallmark of collagen retention and ER stress. The appearance of lamellar body-like structures in cells incubated with the EED inhibitors suggests improved secretory capacity and potentially reflects enhanced trafficking or export of collagen. While the exact nature of these structures remains to be determined, these observations support the hypothesis that PRC2 inhibition improves ER homeostasis and protein handling in OI osteoblasts.

There are still questions that need to be addressed. Our study did not directly measure collagen secretion, folding efficiency, or matrix quality, all of which are central to OI pathophysiology. Also, the precise mechanistic link between PRC2 inhibition and ISR activation remains unclear and needs further investigation of the epigenetic and other mechanisms. In addition, histomorphometry, osteoclast function, and dynamic bone histomorphometry have not yet been evaluated, and the long-term effects and safety of PRC2 inhibition, especially given its role in gene regulation, have yet to be assessed. Another important limitation is the relatively small sample size used for the in vivo studies. Specifically, the mouse experiments were conducted in male G610C mice (*n* = 3 per group), and the effects of EED inhibition in female mice remain to be determined. Future studies will be required to evaluate potential sex-specific differences in treatment response and to further assess the translational potential of this therapeutic approach.

Here, we show that modulation of PRC2 function through the specific inhibition of EED can regulate osteoblast function and ER stress responses in OI. The osteogenic effect of PRC2 appears to be ISR-dependent and provides a basis for targeting intracellular protein processing pathways in OI. Further studies are needed to refine dosing strategies, elucidate downstream mechanisms, and evaluate long-term outcomes before the potential translation of PRC2 inhibition as a therapeutic approach for OI.

## Data Availability

The data that support the findings of this study are available from the corresponding author upon reasonable request.
